# Personalized medicine in rheumatology: the paradigm of serum autoantibodies

**DOI:** 10.1007/s13317-017-0098-1

**Published:** 2017-07-12

**Authors:** Silvia Sirotti, Elena Generali, Angela Ceribelli, Natasa Isailovic, Maria De Santis, Carlo Selmi

**Affiliations:** 1 Division of Rheumatology and Clinical Immunology, Humanitas Research Hospital, Via A. Manzoni 56, Rozzano, 20089 Milan, Italy; 2 BIOMETRA Department, University of Milan, Milan, Italy

**Keywords:** Precision medicine, Tolerance breakdown, Twins, Antinuclear antibodies, Anti-citrullinated peptide antibodies, Rare autoantibodies, Anti-drug antibodies

## Abstract

The sequencing of the human genome is now well recognized as the starting point of personalized medicine. Nonetheless, everyone is unique and can develop different phenotypes of the same disease, despite identical genotypes, as well illustrated by discordant monozygotic twins. To recognize these differences, one of the easiest and most familiar examples of biomarkers capable of identifying and predicting the outcome of patients is represented by serum autoantibodies. In this review, we will describe the concept of personalized medicine and discuss the predictive, prognostic and preventive role of antinuclear antibodies (ANA), anti-citrullinated peptide antibodies (ACPA), rare autoantibodies and anti-drug antibodies (ADA), to evaluate how these can help to identify different disease immune phenotypes and to choose the best option for treating and monitoring rheumatic patients in everyday practice. The importance of ANA resides in the prediction of clinical manifestations in systemic sclerosis and systemic lupus erythematosus and their association with malignancies. ACPA have a predictive role in rheumatoid arthritis, they are associated with the development of a more aggressive disease, extra-articular manifestations and premature mortality in RA patients; moreover, they are capable of predicting therapeutic response. Rare autoantibodies are associated with different disease manifestations and also with a greater incidence of cancer. The determination of ADA levels may be useful in patients where the clinical efficacy of TNF-α inhibitor has dropped, for the assessment of a right management. The resulting scenario supports serum autoantibodies as the cornerstone of personalized medicine in autoimmune diseases.

## Introduction

Following the path of holistic medicine, on the footsteps of Hippocrates, who encouraged his disciples to focus their attention more on “what kind of person has a disease than to know what kind of disease a person has” [1], the human being has reached the goal of a molecular knowledge of itself, revealing the secrets of the genome, through the Human Genome Project completed in 2003. The last decades have seen enormous advances in proteomics, metabolomics and genomics, but the path to the summit is still long [2], as well illustrated by the complexity of the microbiome [3].

From the current stratified medicine, which identifies a group of patients who can benefit from a treatment, medicine is moving toward a personalized approach, whose goal is the right therapy for the right patient at the right time: in other words, a medicine that takes into account individual variability to realize the best preventive and therapeutic strategies [4]. Therapeutic resources for rheumatologic disorders have had a significant development with the advent of biotechnological therapies which have radically changed the course of the disease, the prognosis and the patient’s quality of life. These concepts require a personalized approach based on the risk stratification and on the genetic background of the patient, as well illustrated by Talamonti and colleagues in the case of HLA-Cw06 to predict the response to ustekinumab [5]. Despite these improvements, not all autoimmune diseases respond adequately to therapeutic agents nor we can predict which patients are more susceptible; therefore, it is important to focus on specific preventive measures to identify the ones at risk of developing autoimmune diseases and those who may lead to more severe manifestations.

To realize the purpose of personalized medicine in rheumatology, one of the most useful examples of biomarkers capable of identifying and predicting the outcome of patients, to set appropriate preventative measures, is representing by serum autoantibodies.

## Personalized medicine

Personalized medicine is a system that integrates molecular and biochemical characteristics with patient clinical data, thus introducing the possibility of predicting the disease before the onset of clinical signs and symptoms. It also offers the opportunity to focus on prevention and early intervention, rather than treating advanced stages of diseases and their complications. In this perspective, medical care has to be tailored for each individual, with the aim of offering the best available care for each patient [6, 7].

This approach is part of the “P4” medicine, which not only recognizes the personalized nature of modern medicine, but also expands its horizon to its predictive, preventive and participatory identity [8]. There are numerous advantages in personalizing our medical approach to patients rather than disease, and one of these is the first “P”, that is, prediction. Personalized medicine is expected to subdivide patients into high- and low-risk tiers, based on the integration between genetic factors and biomarkers that provide information on the severity and progression of the diseases [9, 10]. These aspects need to be combined with other factors to increase their predictive value; these include lifestyle (i.e., smoking, obesity), family history for autoimmune disease, genetic profile, clinical manifestation and other laboratory parameters; all of these should be considered as a potential target for prevention and delaying the onset and progression of disease, possibly mediated by additional mechanisms such as the microbiome or epigenetic changes [9, 11]. In this scenario, serum autoantibodies are potentially helpful markers to recognize variants of the same syndrome, enabling to subdivide patients into categories to predict the course of the disease and the possible response to treatment. By excluding patients for whom diagnostic and therapeutic measures are unnecessary, superfluous waste of resources is prevented, resulting in benefits both for the patient and the health-care system [6, 12].

The second “P” stands for prevention, whose purpose is to identify at-risk individuals before the development of clinical manifestations, to implement preventive measures [13]. Traditionally, therapeutic decisions were taken on the basis of physical signs and symptoms observed during medical examination, but frequently these features failed to fully describe diseases. In fact, these signs are often non-specific and subjective; they are also often blurred or absent at the initial stages. So, this approach may miss the opportunity for prevention and early intervention, which is fundamental for the outcome of the patient [6]. Different from this approach, the purpose of personalized medicine is to use biomarkers as screening to identify subjects at high risk of developing disease. This can lead to clinical and economic benefits that come from the prevention of late diagnosis and treatment of complications, resulting in reduced hospitalization for adverse drug reactions and less unnecessary diagnostic interventions. It is not to be underestimated, however, that with the improvement of the quality of life, patients are less likely to use drugs and they would address less frequently to the clinician [9, 12].

The third “P” is to personalize. “Happy families are all alike; every unhappy family is unhappy in its own way”; paraphrasing this Tolstoy’s quote, when people have a problem, they feel different from the others and feel that they do not belong to the average cohort. As a matter of fact, that is not only a subjective perception, but indeed every individual, like the same diseases, are unique, from the genome to environmental factors [14]. In addition, the interpersonal diversity influences the therapeutic response [13]. This variability is elegantly illustrated by monozygotic twins that are largely discordant for autoimmune and chronic inflammatory conditions, thus possibly underlining the effects of non-hereditary factors [15]; hence, each person must be treated as a unique individual [16]. Based on this assumption, pharmaceutical research is shifting from mass therapies to targeted therapies, according to biomarkers that identify those patients who are more likely to successfully respond to treatments [17].

Finally, the fourth “P” indicates the participatory role of personalized medicine; the individual must be involved in the preventive process and personalized treatment to be successful [18]. This main role covered by the individual enriches the model of patient-centered medicine.

We should be aware that autoantibodies represent only a piece (with reasonable costs and within reach for all clinical settings) of a larger scenario and it is obviously unlikely that they are sufficient to achieve personalized medicine.

## The ABC of serum autoantibodies

Autoimmune diseases are estimated to affect nearly 5% of the US population [19] and in the vast majority of cases these are characterized by serum autoantibodies which represent diagnostic markers with, in some cases, a prognostic value and thus in agreement with a personalized management [11, 20], as in the case of paraneoplastic autoimmunity [11]. In general terms, the autoantibody development more frequently precedes the clinical onset of disease [11, 20, 21]. Specific antibody profiles can lead to the identification of disease sub-phenotypes, but only in a few cases the antibody titer correlates with the severity of clinical manifestations or the response to treatment. Therefore, autoantibodies may help the clinician in determining the follow-up and in choosing an appropriate therapy, but might be ineffective in disease severity monitoring [11, 20, 21].

The inconvenient truth of autoantibodies is that these are also detected in a substantial percentage of healthy subjects; thus, their isolated finding has a low positive predictive value which needs to be integrated with other laboratory parameters and patient risk factors [22]. So, it is true that early diagnosis can reduce the economic burden of health care, but the interpretation of serum autoantibodies must be done with proper judgment to avoid inappropriate diagnosis and superfluous treatments. Instead, in subjects with high pre-test probability of an autoimmune disease, the autoantibody positivity is sufficient to set preventative measures such as the elimination of modifiable proposed or established risk factors (smoking, obesity and UV exposure among others) [11] and to warrant a regular rheumatological follow-up.

### Antinuclear antibodies (ANA)

ANA are the most frequently (and often inappropriately) prescribed autoantibody test and they are virtually always positive in connective tissue diseases, i.e., systemic lupus erythematosus (SLE), Sjögren’s syndrome (SjS), scleroderma (SSc), polymyositis and dermatomyositis (PM/DM). The ANA interpretation must thus be driven by clinical suspicion, but their interpretation also depends on both titer and pattern. Different staining patterns, classified into three major groups, nuclear, cytoplasmic and mitotic, give indication on the significance of ANA and type of rheumatic disease (Table 1), and the recent work from the autoantibody standardization group (http://www.ANApatterns.org) provided a long overdue description of the possible profiles. In fact, the gold standard for detecting ANA remains indirect immunofluorescence test (IIF) on HEp-2 cells; however, IIF is time consuming and requires skilled operators to avoid variability. So, new ANA testing strategies have emerged in the last decades, particularly in terms of automation and multiplex analyses. The expected advantages of automated IIF are the reduction of false-negative and false-positive results, the improvement of the uniformity between different readers and laboratories and the efficiency of the evaluation procedure [23]; but unfortunately the sensitivity for rare patterns is not yet adequate and can lead to false-negative results [24, 25].

**Table 1:** IIF ANA patterns and relevant clinical associations

Pattern	Related antigens	Related diagnosis
Nuclear		
Homogeneous	dsDNA, histones, nucleosomes	SLE, drug-induced lupus, JIA
Speckled	hnRNP, U1RNP, Sm, SS-A, SS-B, RNAP-III, Mi-2, Ku	MCTD, SLE, SjS, DM, SSc/PM overlap
Dense fine speckled	DFS70/LEDGF	Rare in SLE, SjS, SSc
Fine speckled	SS-A, SS-B, Mi-2, TIF1γ, TIF1β, Ku, RNA helicase A, replication protein A	SjS, SLE, DM, SSc/PM overlap
Large/coarse speckled	hnRNP, U1RNP, Sm, RNAP-III	MCTD, SLE, SSc
Centromere	CENP-A/B	lcSSc, PBC
Discrete nuclear dots		
Multiple nuclear dots	Sp100, PML protein, MJ/NXP-2	PBC, SARD, PM/DM
Few nuclear dots	p80-coilin, SMN	SjS, SLE, SSc, PM, asymptomatic subjects
Nucleolar		
Homogeneous	PM/Scl75, PM/Scl100, Th/To, B23 nucleophosmin, nucleolin, No55/SC65	SSc, SSc/PM overlap
Clumpy	U3-snoRNP/fibrillarin	SSc
Punctate	RNAP-I, hUBF/NOR-90	SSc, SjS
Nuclear envelope (NE)		
Smooth NE	Lamins A, B, C, or lamin associated proteins	SLE, SjS, seronegative arthritis
Punctate NE	Nuclear pore complex proteins	PBC
Pleomorphic		
PCNA-like	PCNA	SLE, other conditions
CENP F-like	CENP-F	Cancer, other conditions
Cytoplasmic		
Fibrillar		
Linear/actin	Actin, non-muscle myosin	MCTD, CH, cirrhosis, MG, CD, PBC, long-term HD
Filamentous/microtubules	Vimentin, cytokeratin	Infections or inflammations, long-term HD, ALD, SARD, PsO, healthy subjects
Segmental	α actinin, vinculin, tropomyosin	MG, CD, UC
Speckled		
Discrete dots	GW182, Su/Ago2, Ge-1	PBC, SARD, neurological and autoimmune conditions
Dense fine speckled	PL-7, PL-12, ribosomal P proteins	ASS, PM/DM, SLE, juvenile SLE, neuroSLE
Fine speckled	Jo1/histidyl-tRNA synthetase	ASS, PM/DM, lcSSc, IPE
Reticular/AMA	PDC-E2/M2, BCOADC-E2, OGDC-E2, E1α subunit of PDC, E3BP/proteinX	PBC, SSc, rare in other SARD
Polar/Golgi-like	Giantin/macrogolgin, golgin-95/GM130, golgin-160, golgin-97, golgin-245	Rare in SjS, SLE, RA, MCTD, GPA, ICA, PCD, viral infections
Rods and rings	IMPDH2, others	HCV patients post IFN/ribavirin, rare in SLE, Hashimoto’s and healthy controls
Mitotic		
Centrosome	Pericentrin, ninein, Cep250, Cep110, enolase	Rare in SSc, RD, infections (viral and mycoplasma)
Spindle fibers	HsEg5	Rare in SjS, SLE, other SARD
NuMA-like	Centrophilin	SjS, SLE, other
Intercellular bridge	Aurora kinase B, CENP-E, MSA2, KIF14, MKLP1	Rare in SSc, RD, malignancies
Mitotic chromosomal envelope	Modified histone H3, MCA1	Rare in DLE, CLL, SjS, PMR

Table 1 illustrates ANA patterns in IIF in three major categories, subdivided into groups and subgroups of patterns, with their antigenic and diagnostic associations. The information was taken from http://www.ANApattern.org and the related diagnoses were added by Chan EKL et al. [84]

*SLE* systemic lupus erythematosus, *JIA* juvenile idiopathic arthritis, *MCTD* mixed connective tissue disease, *SjS* Sjögren’s syndrome, *DM* dermatomyositis, *PM* polymyositis, *SSc* systemic sclerosis (*lc* limited, *dc* diffuse), *PBC* primary biliary cholangitis, *SARD* systemic autoimmune rheumatic diseases, *CH* chronic hepatitis, *MG* myasthenia gravis, *CD* Crohn’s disease, *HD* haemodialysis, *ALD* alcoholic liver disease, *PsO* psoriasis, *UC* ulcerative colitis, *ASS* anti-synthetase syndrome, *IPE* idiopathic pleural effusion, *RA* rheumatoid arthritis, *GPA* granulomatosis with polyangiitis, *ICA* idiopathic cerebellar ataxia, *PCD* paraneoplastic cerebellar degeneration, *RD* Raynaud’s phenomenon, *DLE* discoid lupus erythematosus, *CLL* chronic lymphocytic leukaemia, *PMR* polymyalgia rheumatica

**Fig. 1:**
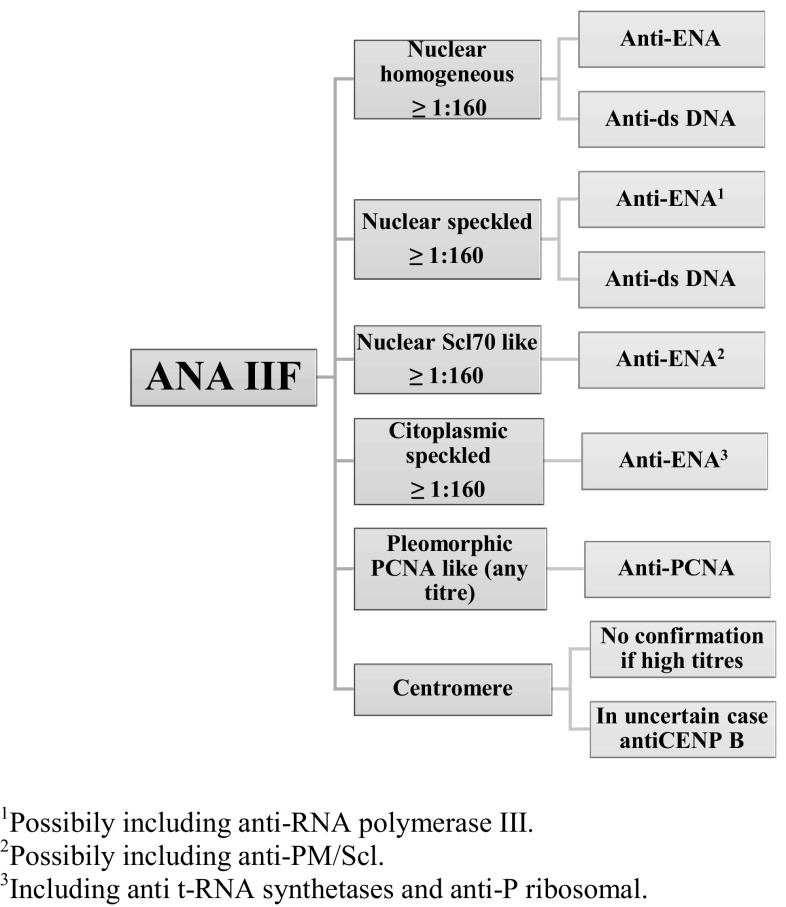
ANA reflex test, modified from Tonutti et al. [31]

It is still unclear what significance ANA may have in asymptomatic patients, since they are not specific markers of connective tissue diseases and can be falsely positive in healthy subjects (the prevalence of ANA in the general population is 13.8%) [26], especially in seniors, as well as in patients with other chronic inflammatory or infectious diseases; otherwise, they can precede clinical manifestations and diagnosis in SSc [27] and SLE [28]. The current recommendations state that ANA-positive subjects should be tested for antibodies to extractable nuclear antigens (anti-ENA) and anti-double-stranded DNA antibodies (anti-dsDNA) [29, 30]. The presence of multiple antibodies thus becomes specific for systemic rheumatic disorder and helps the diagnostic process. To improve the appropriateness of the immunological diagnosis of systemic autoimmune diseases, to accelerate time for completing diagnostic process and to avoid waste of money, the introduction of ANA reflex test has been proposed [31]. The diagnostic algorithm suggested by Tonutti and colleagues begins with a first-line high sensitivity test (i.e., ANA IIF on HEp-2 cells) to allow antibody positivity recognition and the definition of pattern and titer. The second-line tests (high specificity) are done for ANA titers ≥1:160 and include, as mentioned, anti-dsDNA and anti-ENA (by ELISA) to evaluate specific antigenic expression. Figure 1 shows the second-line tests based on the ANA patterns found in IIF.

The predictive significance of ANA has been clearly demonstrated in the seminal work by Arbuckle and colleagues [28]. They studied 130 patients with SLE, whose serum had been collected many years before the diagnosis. Most patients harbored at least one autoantibody up to 9 years before the development of clinical manifestations and therefore diagnosis of SLE, in particular ANA and also antiphospholipid, anti-Ro and anti-La antibodies [32]. The mean time to diagnosis for these autoantibodies was about 3.4 years, while for anti-double-stranded DNA autoantibodies 2.2 years. Later predictors of disease were anti-Sm and anti-nuclear ribonucleoprotein antibodies, which tended to coincide with the onset of signs and symptoms. Another interesting observation is that new types of autoantibodies gradually accumulated before the diagnosis and reached a plateau at the diagnosis. Considering that while ANA, anti-Ro, anti-La and anti-phospholipid antibodies may also be present in healthy subjects, anti-dsDNA, anti-Sm and anti-nuclear ribonucleoprotein antibodies are very rare in the general population. Accordingly, the positivity of these aforementioned autoantibodies should lead to close monitoring.

The value of ANA is not limited to diagnosis, but may have a prognostic role in specific clinical settings such as SSc, where the nucleolar pattern has been associated with a more rapid progression to late scleroderma pattern at nailfold capillaroscopy [33]. Moreover, the presence of abnormal nailfold capillaries with ANA positivity is associated with an increase of mortality in patients with Raynaud’s phenomenon without previously known connective tissue disease exclusively in women [34]. The ANA prognostic value is also reported in juvenile idiopathic arthritis, being associated with the predisposition to uveitis [35].

Finally, ANA and anti-ENA, in particular anti-Scl-70 antibodies, may be associated with malignancies, especially in patients with SSc and inflammatory myositis [36]. Another correlation has been reported with lymphomas, as serum ANA were significantly higher in patients with disease than in controls. In addition, it has been found that the level of lactate dehydrogenase in ANA-positive patients was lower, thus suggesting that these patients have a better prognosis than ANA-negative patients, probably demonstrating the existence of an anti-tumor response that can be associated with a better prognosis [37].

### Anti-citrullinated peptide antibodies (ACPA)

**Fig. 2:**
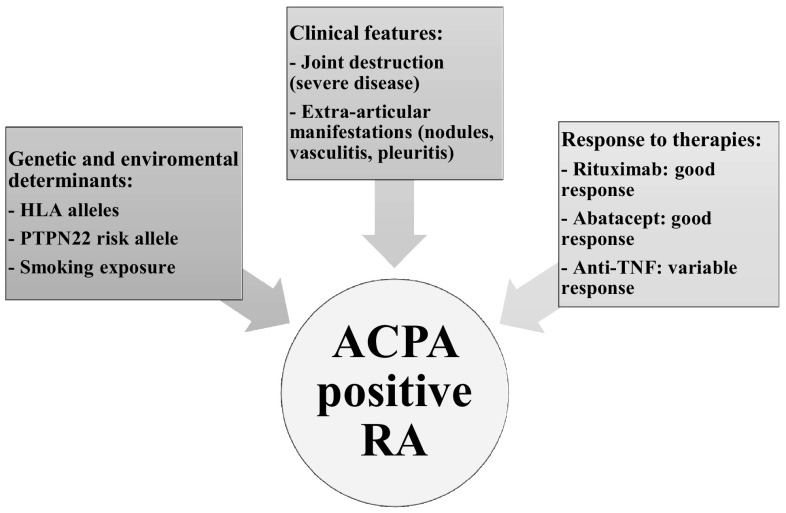
Features of ACPA-positive RA. Modified from Malmstrom et al. [39, 104–114]

ACPA, together with rheumatoid factor (RF), are the characteristic antibodies of rheumatoid arthritis (RA), manifesting higher specificity and lower sensitivity compared to RF for RA. ACPA can divide patients with RA into two subgroups that differ in prognosis and response to treatment [38, 39] (Fig. 2). Patients with ACPA-positive RA differ from seronegative ones in genetics and environmental risk factors. The presence of ACPA is associated with genetic interaction between HLA-DRB1 shared epitope (SE) and PTPN22 risk allele [40], and this points out how MHC class II-dependent T cell activation plays a central pathogenic role in the development of ACPA-positive RA. Furthermore, other gene interactions associated with ACPA-positive RA have been confirmed in several studies [4, 38]. ACPA are highly prevalent not only in patients with HLA-DRB1 SE, but also in those with HLA-DRB1*15 non-SE, proposing that this latter allele can act as a second trigger that may intensify autoimmune response in predisposed subjects [41]. Even gene–environment interaction can determine ACPA-positive RA, particularly in association with smoking and HLA-DRB1 SE, or with smoking and PTPN22 [38]. It is interesting to note that SE and smoking are associated with the presence of all three autoantibodies specifically related with RA [ACPA, RF and anti-carbamylated protein antibodies (anti-CarP)] [42].

The influence of environmental factors, particularly smoking, has been shown to be a risk factor for the development of ACPA-positive RA; other environmental risk factors associated with ACPA are excessive coffee consumption, use of oral contraceptives and periodontitis caused by *Porphyromonas gingivalis*, while moderate alcohol consumption may be protective against this RA subtype [43, 44].

As previously discussed for ANA, also ACPA has a predictive role in autoimmune diseases, as well as RF and anti-RA-33 autoantibodies. In fact, they may occur early in the course of RA and are often present several years before the first symptoms [45, 46].

Numerous studies have shown that the presence of ACPA and their concentration are associated with the development of a more aggressive disease. Positive serum ACPA at baseline leads to a progressing and destructive form of RA, and they are the strongest independent predictor of radiographic damage [47–49]. ACPA have also been correlated with increased inflammation and extra-articular manifestations, high rates of disability and cardiovascular disease, with the latter most responsible for the premature mortality in RA [38].

The distinction between ACPA-positive and ACPA-negative patients can also help us in the therapeutic choice; in fact, the superior efficacy of agents such as rituximab and abatacept in ACPA-positive patients has been demonstrated [50, 51]. This can occur because in these patients, the contribution of B cells to RA pathogenesis is more pronounced, and they might be more responsive to B cell-directed treatments than other patients [52]. Also, methotrexate seems to be more effective in ACPA-positive subjects, in those patients in whom the arthritis stage is not fully defined by the ACR criteria, and it may delay the onset of RA [38]. Conversely, ACPA positivity is correlated with a reduced response to all anti-tumor necrosis factor biologics [53].

### Rare autoantibodies

Rare autoantibodies characterize nearly all rheumatic diseases, despite not being included in any classification criteria set. Due to their low penetrance, they are not used as first-line test or generally available in routine laboratory, but they may help the clinician in making more accurate diagnoses and better management of patients, because they are associated with different disease manifestations and also with a greater incidence of cancer [54]. This is particularly relevant for SSc, inflammatory myositis and SLE, for which we should note that the proposed clinical significance is derived from small cross-sectional studies.

The most significant autoantibodies in SSc are anti-centromere, anti-RNA polymerase III (anti-RNAP) and anti-Scl70, while anti-U3RNP is rarely found. Other autoantibodies are listed in Table 2 with their proposed clinical associations. Anti-RNAP antibodies are related to diffuse cutaneous SSc [55], and they are the most consistent predictors of scleroderma renal crisis [55]. It has also been shown that patients with anti-RNAP antibodies present more solid tumors and that these are temporally associated with the onset of SSc [36, 56]. Anti-Scl-70 antibodies are associated with a higher skin score, disease severity and activity [57], and they are also strong predictors of the development of pulmonary fibrosis [58, 59], digital ulcers [60] and malignancies [61]. Finally, anti-U3RNP antibodies are strongly associated with muscle involvement and with an increased risk of pulmonary arterial hypertension [59, 62, 63].

**Table 2:** Rare autoantibodies in systemic sclerosis and reported clinical associations

Autoantibody	Prevalence (%)	Clinical associations	References
Anti-RNAP	6–31	dcSSc, renal crisis, malignancies	[85, 86]
Anti-Scl70	10–40	dcSSc, ILD, digital ulcers, cardiac involvement, malignancies	[87, 88]
Anti-U3RNP	5–8	dcSSc, cardiomyopathy, myopathy, PAH, ILD, severe small bowel involvement	[89]
Anti-U1RNP	4–14	lcSSc, PAH, overlap syndrome	[90]
Anti-Ku	1–3	Muscle and joint involvement	[91]
Anti-PM-Scl	2–10	myositis, arthritis, lung or kidney involvement and mechanic’s hands	[92]
Anti-Th/To	1–10	lcSSc, PAH, ILD, puffy fingers, small bowel disease, hypothyroidism	[93]
Anti-U11/U12RNP	3	ILD	[94]

*dcSSc* diffuse systemic sclerosis, *lcSSc* limited systemic sclerosis, *PAH* pulmonary arterial hypertension, *PBC* primary biliary cholangitis, *ILD* interstitial lung disease

In the case of PM/DM, serum autoantibodies can be divided into myositis-specific antibodies (MSAs) (anti-Jo1, anti-PL-7, anti-PL-12, anti-EJ, anti-OJ, anti-Mi-2, anti-SRP, anti-KS, anti-TIF1γ/α (anti-155/140), anti-TIF1β, anti-MJ/NXP-2, anti-MDA5/CADM-140, anti-SAE) and myositis-associated antibodies (MAAs) (anti-PM-Scl, anti-Ku, anti-U1-RNP, anti-U1/U2-RNP, anti-U3-RNP) [64]. The most noteworthy clinical associations are reported below, while others are reported in Table 3. In general terms, rare autoantibodies in myositis are used to predict more severe forms, including those associated with cancer as many studies support the possibility that DM and PM are paraneoplastic, particularly DM [65]. Some autoantibodies are associated with major recurrence of neoplasms, and these are anti-TIF1γ/α (anti-155-140) and anti NXP2 antibodies [66, 67]. Jo-1 and other anti-aminoacyl tRNA synthetases (anti-ARS) antibodies (PL-7, PL-12, EJ, OJ, KS) are associated with the so-called anti-synthetase syndrome, characterized by myositis, interstitial lung disease, arthritis, mechanic’s hands and Raynaud’s phenomenon [68, 69]. Anti-Mi-2 antibodies are related to DM with its typical skin manifestation, to good steroid response and good prognosis [70]. Anti-SRP is specific for PM and correlates with a form of necrotizing myopathy, associated with a worse therapeutic response and poor prognosis [64, 71]. Rare autoantibodies detectable in SLE are listed in Table 4; among these, anti-Sm antibodies are associated with lupus nephritis and neuropsychiatric complications, and renal disease association is stronger when they are found together with anti-dsDNA [72]. These antibodies are also associated with discoid lupus and photosensitivity [73]. Anti-ribosomal P antibodies are detected in the cerebrospinal fluid of patients with neuropsychiatric SLE [74], indicating blood–brain barrier permeation, while they are also related to renal [75] and hepatic damages [76], malar rash, oral ulcers and photosensitivity [77].

**Table 3:** Rare autoantibodies in myositis and reported clinical associations. Modified from Satoh et al. [64]

Autoantibody	Prevalence (%)	Clinical associations	References
Anti-ARS	1–30	Anti-synthetase syndrome	[69, 95]
Anti-Mi2	10	DM with typical skin lesions, mild disease	[69, 70, 95]
Anti-TIF1γ	10–15	Severe DM, malignancies	[70, 95]
Anti-NPX2	1–5	Severe DM, severe skin disease, malignancies	[69, 70, 95]
Anti-MDA5	15–20	CADM, ILD, severe skin manifestations, poor prognosis	[69, 95, 96]
Anti-SAE	1	Amyopathic DM	[95]
Anti-SRP	5	Necrotizing myopathy	[70]
Anti-HMGCR	6	Necrotizing myopathy, proximal muscle weakness, elevated CK levels, prior statin use	[70, 95, 97]

*Anti-ARS* anti-aminoacyl tRNA synthetases (Jo1, PL-7, PL-12, EJ, OJ, KS), *DM* dermatomyositis, *CADM* clinically amyopathic dermatomyositis, *ILD* interstitial lung disease, *CK* creatine kinase

**Table 4:** Rare autoantibodies in systemic lupus erythematosus and reported associations

Autoantibody	Prevalence (%)	Clinical associations	References
Anti-Sm	15	LN, NPSLE, discoid SLE, photosensitivity	[98, 99]
Anti-ribosomal P	10	NPSLE, renal and hepatic disorders, malar rash, oral ulcer and photosensitivity, blood–brain barrier permeation	[77, 98]
Anti-La (SS-B)	10–15	Organ dysfunction (kidney, lung, liver)	[100, 101]
Anti-Ki	6–20	Hashimoto’s thyroiditis, ITP, idiopathic ILD, PM/SSc overlap, synovitis, pericarditis, PAH, skin involvement and sicca symptoms	[102]
Anti-histone	21–81	Drug-induced SLE	[103]

*LN* lupus nephritis, *NPSLE* neuro psychiatric SLE, *SCLE* subacute cutaneous SLE, *UCTD* undifferentiated connective tissue disease, *ITP* autoimmune thrombocytopenia

### Anti-drug antibodies (ADA)

Since the introduction of biological drugs, the treatment of immune-mediated inflammatory diseases has witnessed an enormous improvement in the clinical armamentarium. Indeed, tumor necrosis factor α (TNF-α) inhibitors neutralizes the cytokine pro-inflammatory effect. Infliximab (IFX), adalimumab (ADL), golimumab (GOL), etanercept (ETA) and certolizumab (CTZ) can induce an immune response with the formation of autoantibodies against the drug, i.e., elicit immunogenicity, particularly after their prolonged use [78, 79].

ADA formation is caused by the recognition of drugs as non-self substances by the immune system. ADA ultimately act by causing the formation of complexes that block the binding between the drug and the target and also enhance the drug’s clearance [78]. Antibodies thus lead to a reduction in the serum levels of the drug to sub-therapeutic levels, resulting in loss of clinical efficacy [79].

**Fig. 3:**
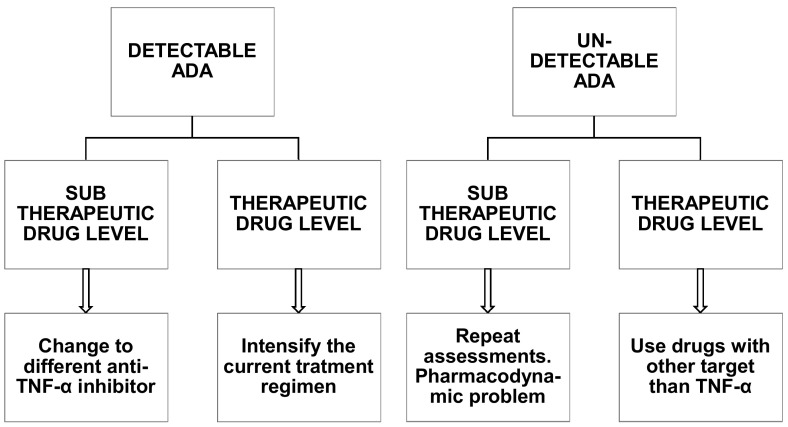
Proposed algorithm in patients with a rheumatic disease and anti-TNF-α treatment failure. Modified from Steenholdt et al. [80]

Therapeutic drug monitoring, done by measuring the TNF-α inhibitors serum levels and ADA, is considered an important factor for personalized TNF-α inhibitor treatment in chronic autoimmune diseases [80]. Nevertheless, the mere presence of ADA does not directly correlate to drug loss of response and clinical consequences, since a significant amount of ADA is needed to neutralize much of the serum drug, which is usually given in high doses. More importantly, there is no agreement on the laboratory methods to be used for ADA detection in clinical practice. The significance of ADA largely depends on the quantity of antibodies produced and on the amount of drug not bound to them; if the quantity of ADA-free drug is substantial, the clinical impact will be minimal [81]. Several studies have shown that ADA become detectable especially within the first year of treatment with TNF-α inhibitors, while their immunogenicity is very low after the first year of therapy [82, 83]. ADA levels are diminished using maintenance therapy rather than episodic therapy, since prolonged periods in which the drug is sub-dosed are avoided [80]. Moreover, patients treated concomitantly with MTX have a lower risk of developing such antibodies, similarly to those responding to therapy [78]. Overall, the determination of ADA levels and anti-TNF-α drug may be useful in patients where the clinical efficacy of TNF-α inhibitor has dropped. Pecoraro and colleagues suggest that in the presence of high ADA levels, either with optimal or suboptimal drug levels, the anti-TNF-α should be changed, while, in the case of low ADA levels, if drug levels are not adequate, the dosage or frequency of drug administration should be increased. Ultimately, however, the secondary loss of efficacy of an anti-TNF-α drug will lead to a treatment switch regardless of the mechanisms responsible for such loss. However others, in particular Steenholdt, suggest a different approach as shown in Fig. 3.

## Conclusions

The technological advances and a thorough knowledge of diseases pathogenesis over the last decade, combined with the increasing expectations of patients, have laid the foundation for developing an individualized management for a single patient, as opposed to that from the ‘one size fits all’ older school approach. The benefits of this approach have been discussed here, but they underline the numerous unmet needs in this field. In the case of serum autoantibodies, we cannot overlook the technological and methodological limitations that preclude (or prevent from) conclusive evidence. We would need more sensitive methods and experienced operators for the identification of rare antibodies and patterns. Moreover, most hospitals do not have laboratories with adequate machinery for rare autoantibodies or lack adequate standardization.

In general terms, the information derived from antibody positivity is of great help in setting up the most appropriate management, but it does not give us the assurance that the disease will be more severe, that the patients will develop organ involvement or even that they will develop malignancies. Because they are not specific tests for these manifestations, their positivity should act as a clue that draws our attention to such possible conditions. Their superficial interpretation could lead to excessive and useless diagnostic procedures and over-treatment, also causing unnecessary concern to the patient and his family.

These data, therefore, must be evaluated critically and integrated with the clinical history of the patient and with other laboratory data resulting in an a priori probability of having a specific disease. In an ancient Indian tale, several blind men seek to learn the nature of an elephant. One who examines the trunk describes the elephant to be like a snake. Another who feels one of the powerful legs states that the animal is similar to a tree trunk. The last, having touched the large elephant’s ear, believes that this animal might be able to fly. Each blind man, focusing only on that part they were able to examine, missed entirely the true shape of the elephant. Understanding the individual components of the biological complexity of subjects is an important first step, but to better appreciate the “big picture,” avoiding the mistake of those blind men is necessary to integrate all the information that comes from the patient as a unique individual, different from any other, so that the intent of personalized medicine can be realized.

## References

[1] Murugan R (2015). Movement towards personalised medicine in the ICU. Lancet Respir Med.

[2] Collins FS, Varmus H (2015). A new initiative on precision medicine. N Engl J Med.

[3] Tropini C, Earle KA, Huang KC, Sonnenburg JL (2017). The Gut microbiome: connecting spatial organization to function. Cell Host Microbe.

[4] Goulielmos GN, Zervou MI, Myrthianou E, Burska A, Niewold TB, Ponchel F (2016). Genetic data: the new challenge of personalized medicine, insights for rheumatoid arthritis patients. Gene.

[5] Talamonti M, Botti E, Galluzzo M, Teoli M, Spallone G, Bavetta M, Chimenti S, Costanzo A (2013). Pharmacogenetics of psoriasis: hLA-Cw6 but not LCE3B/3C deletion nor TNFAIP3 polymorphism predisposes to clinical response to interleukin 12/23 blocker ustekinumab. Br J Dermatol.

[6] National Research Council (2011). Toward precision medicine: building a knowledge network for biomedical research and a new taxonomy of disease. National Academies Press, Washington, DC. doi:10.17226/13284 22536618

[7] The Lancet (2011). Moving toward precision medicine. Lancet 378 (9804):1678. doi:10.1016/S0140-6736(11)61725-X 10.1016/S0140-6736(11)61725-X 22078672

[8] Younesi E, Hofmann-Apitius M (2013). From integrative disease modeling to predictive, preventive, personalized and participatory (P4) medicine. EPMA J.

[9] Whitcomb DC (2012). What is personalized medicine and what should it replace?. Nat Rev Gastroenterol Hepatol.

[10] Mayeux R (2004). Biomarkers: potential uses and limitations. NeuroRx.

[11] Damoiseaux J, Andrade LE, Fritzler MJ, Shoenfeld Y (2015). Autoantibodies 2015: from diagnostic biomarkers toward prediction, prognosis and prevention. Autoimmun Rev.

[12] Abrahams E, Ginsburg GS, Silver M (2005). The personalized medicine coalition: goals and strategies. Am J Pharmacogenom.

[13] Hood L (2013). Systems biology and p4 medicine: past, present, and future. Rambam Maimonides Med J.

[14] Floreani A, Leung PS, Gershwin ME (2016). Environmental basis of autoimmunity. Clin Rev Allergy Immunol.

[15] Generali E, Ceribelli A, Stazi MA, Selmi C (2017). Lessons learned from twins in autoimmune and chronic inflammatory diseases. J Autoimmun.

[16] Hood L, Flores M (2012). A personal view on systems medicine and the emergence of proactive P4 medicine: predictive, preventive, personalized and participatory. N Biotechnol.

[17] Nohaile M (2011). The biomarker is not the end. Drug Discov Today.

[18] Sagner M, McNeil A, Puska P, Auffray C, Price ND, Hood L, Lavie CJ, Han ZG, Chen Z, Brahmachari SK, McEwen BS, Soares MB, Balling R, Epel E, Arena R (2017). The P4 health spectrum—a predictive, preventive, personalized and participatory continuum for promoting healthspan. Prog Cardiovasc Dis.

[19] Walsh SJ, Rau LM (2000). Autoimmune diseases: a leading cause of death among young and middle-aged women in the United States. Am J Public Health.

[20] Harel M, Shoenfeld Y (2006). Predicting and preventing autoimmunity, myth or reality?. Ann N Y Acad Sci.

[21] Bizzaro N, Tozzoli R, Shoenfeld Y (2007). Are we at a stage to predict autoimmune rheumatic diseases?. Arthritis Rheum.

[22] Selmi C, Ceribelli A, Generali E, Scire CA, Alborghetti F, Colloredo G, Porrati L, Achenza MI, De Santis M, Cavaciocchi F, Massarotti M, Isailovic N, Paleari V, Invernizzi P, Matthias T, Zucchi A, Meroni PL (2016). Serum antinuclear and extractable nuclear antigen antibody prevalence and associated morbidity and mortality in the general population over 15 years. Autoimmun Rev.

[23] Tozzoli R, Antico A, Porcelli B, Bassetti D (2012). Automation in indirect immunofluorescence testing: a new step in the evolution of the autoimmunology laboratory. Auto Immun Highlights.

[24] Op De Beeck K, Vermeersch P, Verschueren P, Westhovens R, Marien G, Blockmans D, Bossuyt X (2012). Antinuclear antibody detection by automated multiplex immunoassay in untreated patients at the time of diagnosis. Autoimmun Rev.

[25] Litwin CM, Binder SR (2016). ANA testing in the presence of acute and chronic infections. J Immunoassay Immunochem.

[26] Satoh M, Chan EK, Ho LA, Rose KM, Parks CG, Cohn RD, Jusko TA, Walker NJ, Germolec DR, Whitt IZ, Crockett PW, Pauley BA, Chan JY, Ross SJ, Birnbaum LS, Zeldin DC, Miller FW (2012). Prevalence and sociodemographic correlates of antinuclear antibodies in the United States. Arthritis Rheum.

[27] Kuwana M (2017). Circulating anti-nuclear antibodies in systemic sclerosis: utility in diagnosis and disease subsetting. J Nippon Med Sch.

[28] Arbuckle MR, McClain MT, Rubertone MV, Scofield RH, Dennis GJ, James JA, Harley JB (2003). Development of autoantibodies before the clinical onset of systemic lupus erythematosus. N Engl J Med.

[29] Tozzoli R, Villalta D, Bizzaro N (2016). Challenges in the standardization of autoantibody testing: a comprehensive review. Clin Rev Allergy Immunol.

[30] Bizzaro N, Wiik A (2004). Appropriateness in anti-nuclear antibody testing: from clinical request to strategic laboratory practice. Clin Exp Rheumatol.

[31] Tonutti E, Bizzaro N, Morozzi G, Radice A, Cinquanta L, Villalta D, Tozzoli R, Tampoia M, Porcelli B, Fabris M, Brusca I, Alessio MG, Barberio G, Sorrentino MC, Antico A, Bassetti D, Fontana DE, Imbastaro T, Visentini D, Pesce G, Bagnasco M, Study Group on Autoimmune Diseases of the Italian Society of Clinical P, Laboratory M (2016). The ANA-reflex test as a model for improving clinical appropriateness in autoimmune diagnostics. Auto Immun Highlights.

[32] Satoh M, Yamagata H, Watanabe F, Nakayama S, Ogasawara T, Tojo T, Akizuki M (1995). Development of anti-Sm and anti-DNA antibodies followed by clinical manifestation of systemic lupus erythematosus in an elderly woman with long-standing Sjogren’s syndrome. Lupus.

[33] Sulli A, Ruaro B, Smith V, Pizzorni C, Zampogna G, Gallo M, Cutolo M (2013). Progression of nailfold microvascular damage and antinuclear antibody pattern in systemic sclerosis. J Rheumatol.

[34] Mueller M, Gschwandtner ME, Gamper J, Giurgea GA, Charwat-Resl S, Kiener HP, Smolen JS, Perkmann T, Koppensteiner R, Schlager O (2016). Relation of nailfold capillaries and autoantibodies to mortality in patients with raynaud phenomenon. Circulation.

[35] Swart JF, de Roock S, Prakken BJ (2016). Understanding inflammation in juvenile idiopathic arthritis: how immune biomarkers guide clinical strategies in the systemic onset subtype. Eur J Immunol.

[36] Shah AA, Hummers LK, Casciola-Rosen L, Visvanathan K, Rosen A, Wigley FM (2015). Examination of autoantibody status and clinical features associated with cancer risk and cancer-associated scleroderma. Arthritis Rheumatol.

[37] Zou HY, Gu X, Yu WZ, Wang Z, Jiao M (2015). Detection of serum antinuclear antibodies in lymphoma patients. Genet Mol Res.

[38] Klareskog L, Catrina AI, Paget S (2009). Rheumatoid arthritis. Lancet.

[39] Malmstrom V, Catrina AI, Klareskog L (2017). The immunopathogenesis of seropositive rheumatoid arthritis: from triggering to targeting. Nat Rev Immunol.

[40] Kurko J, Besenyei T, Laki J, Glant TT, Mikecz K, Szekanecz Z (2013). Genetics of rheumatoid arthritis - a comprehensive review. Clin Rev Allergy Immunol.

[41] Laki J, Lundstrom E, Snir O, Ronnelid J, Ganji I, Catrina AI, Bengtsson C, Saevarsdottir S, Wick MC, Alfredsson L, Klareskog L, Padyukov L (2012). Very high levels of anti-citrullinated protein antibodies are associated with HLA-DRB1*15 non-shared epitope allele in patients with rheumatoid arthritis. Arthritis Rheum.

[42] Gan RW, Trouw LA, Shi J, Toes RE, Huizinga TW, Demoruelle MK, Kolfenbach JR, Zerbe GO, Deane KD, Edison JD, Gilliland WR, Norris JM, Holers VM (2015). Anti-carbamylated protein antibodies are present prior to rheumatoid arthritis and are associated with its future diagnosis. J Rheumatol.

[43] Pedersen M, Jacobsen S, Klarlund M, Pedersen BV, Wiik A, Wohlfahrt J, Frisch M (2006). Environmental risk factors differ between rheumatoid arthritis with and without auto-antibodies against cyclic citrullinated peptides. Arthritis Res Ther.

[44] Detert J, Pischon N, Burmester GR, Buttgereit F (2010). The association between rheumatoid arthritis and periodontal disease. Arthritis Res Ther.

[45] Nell VP, Machold KP, Stamm TA, Eberl G, Heinzl H, Uffmann M, Smolen JS, Steiner G (2005). Autoantibody profiling as early diagnostic and prognostic tool for rheumatoid arthritis. Ann Rheum Dis.

[46] Muller S, Radic M (2015). Citrullinated autoantigens: from diagnostic markers to pathogenetic mechanisms. Clin Rev Allergy Immunol.

[47] Bukhari M, Thomson W, Naseem H, Bunn D, Silman A, Symmons D, Barton A (2007). The performance of anti-cyclic citrullinated peptide antibodies in predicting the severity of radiologic damage in inflammatory polyarthritis: results from the Norfolk Arthritis Register. Arthritis Rheum.

[48] Visser K, Goekoop-Ruiterman YP, de Vries-Bouwstra JK, Ronday HK, Seys PE, Kerstens PJ, Huizinga TW, Dijkmans BA, Allaart CF (2010). A matrix risk model for the prediction of rapid radiographic progression in patients with rheumatoid arthritis receiving different dynamic treatment strategies: post hoc analyses from the BeSt study. Ann Rheum Dis.

[49] Jilani AA, Mackworth-Young CG (2015). The role of citrullinated protein antibodies in predicting erosive disease in rheumatoid arthritis: a systematic literature review and meta-analysis. Int J Rheumatol.

[50] Silverman GJ, Pelzek A (2014). Rheumatoid arthritis clinical benefits from abatacept, cytokine blockers, and rituximab are all linked to modulation of memory B cell responses. J Rheumatol.

[51] Gottenberg JE, Cinquetti G, Larroche C, Combe B, Hachulla E, Meyer O, Pertuiset E, Kaplanski G, Chiche L, Berthelot JM, Gombert B, Goupille P, Marcelli C, Feuillet S, Leone J, Sibilia J, Zarnitsky C, Carli P, Rist S, Gaudin P, Salliot C, Piperno M, Deplas A, Breban M, Lequerre T, Richette P, Ghiringhelli C, Hamidou M, Ravaud P, Mariette X, Club Rhumatismes et I, the French Society of R (2013). Efficacy of rituximab in systemic manifestations of primary Sjogren’s syndrome: results in 78 patients of the AutoImmune and Rituximab registry. Ann Rheum Dis.

[52] Schreiber K, Nocturne G, Cornec D, Daien CI (2017). Lymphocytes as biomarkers of therapeutic response in rheumatic autoimmune diseases, is it a realistic goal?. Clin Rev Allergy Immunol.

[53] Potter C, Hyrich KL, Tracey A, Lunt M, Plant D, Symmons DP, Thomson W, Worthington J, Emery P, Morgan AW, Wilson AG, Isaacs J, Barton A, Braggss (2009). Association of rheumatoid factor and anti-cyclic citrullinated peptide positivity, but not carriage of shared epitope or PTPN22 susceptibility variants, with anti-tumour necrosis factor response in rheumatoid arthritis. Ann Rheum Dis.

[54] Gunawardena H (2017). The clinical features of myositis-associated autoantibodies: a review. Clin Rev Allergy Immunol.

[55] Bunn CC, Denton CP, Shi-Wen X, Knight C, Black CM (1998). Anti-RNA polymerases and other autoantibody specificities in systemic sclerosis. Br J Rheumatol.

[56] Moinzadeh P, Fonseca C, Hellmich M, Shah AA, Chighizola C, Denton CP, Ong VH (2014). Association of anti-RNA polymerase III autoantibodies and cancer in scleroderma. Arthritis Res Ther.

[57] Hu PQ, Fertig N, Medsger TA, Wright TM (2003). Correlation of serum anti-DNA topoisomerase I antibody levels with disease severity and activity in systemic sclerosis. Arthritis Rheum.

[58] Henault J, Robitaille G, Senecal JL, Raymond Y (2006). DNA topoisomerase I binding to fibroblasts induces monocyte adhesion and activation in the presence of anti-topoisomerase I autoantibodies from systemic sclerosis patients. Arthritis Rheum.

[59] Le Pavec J, Launay D, Mathai SC, Hassoun PM, Humbert M (2011). Scleroderma lung disease. Clin Rev Allergy Immunol.

[60] Walker UA, Tyndall A, Czirjak L, Denton C, Farge-Bancel D, Kowal-Bielecka O, Muller-Ladner U, Bocelli-Tyndall C, Matucci-Cerinic M (2007). Clinical risk assessment of organ manifestations in systemic sclerosis: a report from the EULAR Scleroderma Trials And Research group database. Ann Rheum Dis.

[61] Shah AA, Casciola-Rosen L, Rosen A (2015). Review: cancer-induced autoimmunity in the rheumatic diseases. Arthritis Rheumatol.

[62] Hamaguchi Y (2010). Autoantibody profiles in systemic sclerosis: predictive value for clinical evaluation and prognosis. J Dermatol.

[63] Steen VD (2005). Autoantibodies in systemic sclerosis. Semin Arthritis Rheum.

[64] Satoh M, Tanaka S, Ceribelli A, Calise SJ, Chan EK (2017). A Comprehensive overview on myositis-specific antibodies: new and old biomarkers in idiopathic inflammatory myopathy. Clin Rev Allergy Immunol.

[65] Tiniakou E, Mammen AL (2017). Idiopathic inflammatory myopathies and malignancy: a comprehensive review. Clin Rev Allergy Immunol.

[66] Ceribelli A, Isailovic N, De Santis M, Generali E, Fredi M, Cavazzana I, Franceschini F, Cantarini L, Satoh M, Selmi C (2017). Myositis-specific autoantibodies and their association with malignancy in Italian patients with polymyositis and dermatomyositis. Clin Rheumatol.

[67] Chinoy H, Fertig N, Oddis CV, Ollier WE, Cooper RG (2007). The diagnostic utility of myositis autoantibody testing for predicting the risk of cancer-associated myositis. Ann Rheum Dis.

[68] Ascherman DP (2015). Role of Jo-1 in the Immunopathogenesis of the Anti-synthetase Syndrome. Curr Rheumatol Rep.

[69] Mammen AL (2011). Autoimmune myopathies: autoantibodies, phenotypes and pathogenesis. Nat Rev Neurol.

[70] Casciola-Rosen L, Mammen AL (2012). Myositis autoantibodies. Curr Opin Rheumatol.

[71] Aggarwal R, Oddis CV, Goudeau D, Fertig N, Metes I, Stephens C, Qi Z, Koontz D, Levesque MC (2015). Anti-signal recognition particle autoantibody ELISA validation and clinical associations. Rheumatology (Oxford).

[72] Migliorini P, Baldini C, Rocchi V, Bombardieri S (2005). Anti-Sm and anti-RNP antibodies. Autoimmunity.

[73] Fredi M, Cavazzana I, Quinzanini M, Taraborelli M, Cartella S, Tincani A, Franceschini F (2014). Rare autoantibodies to cellular antigens in systemic lupus erythematosus. Lupus.

[74] West SG, Emlen W, Wener MH, Kotzin BL (1995). Neuropsychiatric lupus erythematosus: a 10-year prospective study on the value of diagnostic tests. Am J Med.

[75] Martin AL, Reichlin M (1996). Fluctuations of antibody to ribosomal P proteins correlate with appearance and remission of nephritis in SLE. Lupus.

[76] Koren E, Schnitz W, Reichlin M (1993). Concomitant development of chronic active hepatitis and antibodies to ribosomal P proteins in a patient with systemic lupus erythematosus. Arthritis Rheum.

[77] Shi ZR, Cao CX, Tan GZ, Wang L (2015). The association of serum anti-ribosomal P antibody with clinical and serological disorders in systemic lupus erythematosus: a systematic review and meta-analysis. Lupus.

[78] Pecoraro V, De Santis E, Melegari A, Trenti T (2017). The impact of immunogenicity of TNFalpha inhibitors in autoimmune inflammatory disease. A systematic review and meta-analysis. Autoimmun Rev.

[79] Moots RJ, Xavier RM, Mok CC, Rahman MU, Tsai WC, Al-Maini MH, Pavelka K, Mahgoub E, Kotak S, Korth-Bradley J, Pedersen R, Mele L, Shen Q, Vlahos B (2017). The impact of anti-drug antibodies on drug concentrations and clinical outcomes in rheumatoid arthritis patients treated with adalimumab, etanercept, or infliximab: results from a multinational, real-world clinical practice, non-interventional study. PLoS One.

[80] Steenholdt C (2016). Personalized therapy with TNF-inhibitors in Crohn’s disease: optimizing treatment outcomes by monitoring drug levels and anti-drug antibodies. Dan Med J.

[81] Bloem K, Hernandez-Breijo B, Martinez-Feito A, Rispens T (2017). Immunogenicity of therapeutic antibodies: monitoring anti-drug antibodies in a clinical context. Ther Drug Monit.

[82] Baert F, Noman M, Vermeire S, Van Assche G, Haens GD, Carbonez A, Rutgeerts P (2003). Influence of immunogenicity on the long-term efficacy of infliximab in Crohn’s disease. N Engl J Med.

[83] Ungar B, Chowers Y, Yavzori M, Picard O, Fudim E, Har-Noy O, Kopylov U, Eliakim R, Ben-Horin S, Consortium A (2014). The temporal evolution of antidrug antibodies in patients with inflammatory bowel disease treated with infliximab. Gut.

[84] Chan EK, Damoiseaux J, Carballo OG, Conrad K, de Melo Cruvinel W, Francescantonio PL, Fritzler MJ, Garcia-De La Torre I, Herold M, Mimori T, Satoh M, von Muhlen CA, Andrade LE (2015). Report of the first international consensus on standardized nomenclature of antinuclear antibody HEp-2 cell patterns 2014–2015. Front Immunol.

[85] Sobanski V, Dauchet L, Lefevre G, Lambert M, Morell-Dubois S, Sy T, Hachulla E, Hatron PY, Launay D, Dubucquoi S (2014). Prevalence of anti-RNA polymerase III antibodies in systemic sclerosis: new data from a French cohort and a systematic review and meta-analysis. Arthritis Rheumatol.

[86] Kayser C, Fritzler MJ (2015). Autoantibodies in systemic sclerosis: unanswered questions. Front Immunol.

[87] Villalta D, Imbastaro T, Di Giovanni S, Lauriti C, Gabini M, Turi MC, Bizzaro N (2012). Diagnostic accuracy and predictive value of extended autoantibody profile in systemic sclerosis. Autoimmun Rev.

[88] Reveille JD, Solomon DH, American College of Rheumatology Ad Hoc Committee of Immunologic Testing G (2003). Evidence-based guidelines for the use of immunologic tests: anticentromere, Scl-70, and nucleolar antibodies. Arthritis Rheum.

[89] Aggarwal R, Lucas M, Fertig N, Oddis CV, Medsger TA (2009). Anti-U3 RNP autoantibodies in systemic sclerosis. Arthritis Rheum.

[90] Ihn H, Yamane K, Yazawa N, Kubo M, Fujimoto M, Sato S, Kikuchi K, Tamaki K (1999). Distribution and antigen specificity of anti-U1RNP antibodies in patients with systemic sclerosis. Clin Exp Immunol.

[91] Rozman B, Cucnik S, Sodin-Semrl S, Czirjak L, Varju C, Distler O, Huscher D, Aringer M, Steiner G, Matucci-Cerinic M, Guiducci S, Stamenkovic B, Stankovic A, Kveder T (2008). Prevalence and clinical associations of anti-Ku antibodies in patients with systemic sclerosis: a European EUSTAR-initiated multi-centre case-control study. Ann Rheum Dis.

[92] Mahler M, Fritzler MJ (2009). The changing landscape of the clinical value of the PM/Scl autoantibody system. Arthritis Res Ther.

[93] Mitri GM, Lucas M, Fertig N, Steen VD, Medsger TA (2003). A comparison between anti-Th/To- and anticentromere antibody-positive systemic sclerosis patients with limited cutaneous involvement. Arthritis Rheum.

[94] Fertig N, Domsic RT, Rodriguez-Reyna T, Kuwana M, Lucas M, Medsger TA, Feghali-Bostwick CA (2009). Anti-U11/U12 RNP antibodies in systemic sclerosis: a new serologic marker associated with pulmonary fibrosis. Arthritis Rheum.

[95] Ghirardello A, Borella E, Beggio M, Franceschini F, Fredi M, Doria A (2014). Myositis autoantibodies and clinical phenotypes. Auto Immun Highlights.

[96] Fiorentino D, Chung L, Zwerner J, Rosen A, Casciola-Rosen L (2011). The mucocutaneous and systemic phenotype of dermatomyositis patients with antibodies to MDA5 (CADM-140): a retrospective study. J Am Acad Dermatol.

[97] Mammen AL, Chung T, Christopher-Stine L, Rosen P, Rosen A, Doering KR, Casciola-Rosen LA (2011). Autoantibodies against 3-hydroxy-3-methylglutaryl-coenzyme A reductase in patients with statin-associated autoimmune myopathy. Arthritis Rheum.

[98] Satoh M, Vazquez-Del Mercado M, Chan EK (2009). Clinical interpretation of antinuclear antibody tests in systemic rheumatic diseases. Mod Rheumatol.

[99] Flechsig A, Rose T, Barkhudarova F, Strauss R, Klotsche J, Dahnrich C, Schlumberger W, Enghard P, Burmester GR, Hiepe F, Biesen R (2017) What is the clinical significance of anti-Sm antibodies in systemic lupus erythematosus? A comparison with anti-dsDNA antibodies and C3. Clin Exp Rheumatol [**Epub ahead of print**] 28281463

[100] Routsias JG, Tzioufas AG (2010). B-cell epitopes of the intracellular autoantigens Ro/SSA and La/SSB: tools to study the regulation of the autoimmune response. J Autoimmun.

[101] Novak GV, Marques M, Balbi V, Gormezano NW, Kozu K, Sakamoto AP, Pereira RM, Terreri MT, Magalhaes CS, Guariento A, Sallum AM, Marini R, Ferriani VP, Barbosa CM, de Castro TC, Ramos VC, Bonfa E, Silva CA (2017). Anti-RO/SSA and anti-La/SSB antibodies: association with mild lupus manifestations in 645 childhood-onset systemic lupus erythematosus. Autoimmun Rev.

[102] Cavazzana I, Franceschini F, Vassalini C, Danieli E, Quinzanini M, Airo P, Cattaneo R (2005). Clinical and serological features of 35 patients with anti-Ki autoantibodies. Lupus.

[103] Cohen MG, Pollard KM, Webb J (1992). Antibodies to histones in systemic lupus erythematosus: prevalence, specificity, and relationship to clinical and laboratory features. Ann Rheum Dis.

[104] Klareskog L, Stolt P, Lundberg K, Kallberg H, Bengtsson C, Grunewald J, Ronnelid J, Harris HE, Ulfgren AK, Rantapaa-Dahlqvist S, Eklund A, Padyukov L, Alfredsson L (2006). A new model for an etiology of rheumatoid arthritis: smoking may trigger HLA-DR (shared epitope)-restricted immune reactions to autoantigens modified by citrullination. Arthritis Rheum.

[105] Huizinga TW, Amos CI, van der Helm-van Mil AH, Chen W, van Gaalen FA, Jawaheer D, Schreuder GM, Wener M, Breedveld FC, Ahmad N, Lum RF, de Vries RR, Gregersen PK, Toes RE, Criswell LA (2005). Refining the complex rheumatoid arthritis phenotype based on specificity of the HLA-DRB1 shared epitope for antibodies to citrullinated proteins. Arthritis Rheum.

[106] Kallberg H, Padyukov L, Plenge RM, Ronnelid J, Gregersen PK, van der Helm-van Mil AH, Toes RE, Huizinga TW, Klareskog L, Alfredsson L, Epidemiological Investigation of Rheumatoid Arthritis study g (2007). Gene-gene and gene-environment interactions involving HLA-DRB1, PTPN22, and smoking in two subsets of rheumatoid arthritis. Am J Hum Genet.

[107] Viatte S, Plant D, Bowes J, Lunt M, Eyre S, Barton A, Worthington J (2012). Genetic markers of rheumatoid arthritis susceptibility in anti-citrullinated peptide antibody negative patients. Ann Rheum Dis.

[108] Linn-Rasker SP, van der Helm-van Mil AH, van Gaalen FA, Kloppenburg M, de Vries RR, le Cessie S, Breedveld FC, Toes RE, Huizinga TW (2006). Smoking is a risk factor for anti-CCP antibodies only in rheumatoid arthritis patients who carry HLA-DRB1 shared epitope alleles. Ann Rheum Dis.

[109] Willemze A, Trouw LA, Toes RE, Huizinga TW (2012). The influence of ACPA status and characteristics on the course of RA. Nat Rev Rheumatol.

[110] Turesson C, Jacobsson LT, Sturfelt G, Matteson EL, Mathsson L, Ronnelid J (2007). Rheumatoid factor and antibodies to cyclic citrullinated peptides are associated with severe extra-articular manifestations in rheumatoid arthritis. Ann Rheum Dis.

[111] Korkmaz C, Us T, Kasifoglu T, Akgun Y (2006). Anti-cyclic citrullinated peptide (CCP) antibodies in patients with long-standing rheumatoid arthritis and their relationship with extra-articular manifestations. Clin Biochem.

[112] Martin-Mola E, Balsa A, Garcia-Vicuna R, Gomez-Reino J, Gonzalez-Gay MA, Sanmarti R, Loza E (2016). Anti-citrullinated peptide antibodies and their value for predicting responses to biologic agents: a review. Rheumatol Int.

[113] Sokolove J, Schiff M, Fleischmann R, Weinblatt ME, Connolly SE, Johnsen A, Zhu J, Maldonado MA, Patel S, Robinson WH (2016). Impact of baseline anti-cyclic citrullinated peptide-2 antibody concentration on efficacy outcomes following treatment with subcutaneous abatacept or adalimumab: 2-year results from the AMPLE trial. Ann Rheum Dis.

[114] Smolen JS, Burmester GR, Combe B, Curtis JR, Hall S, Haraoui B, van Vollenhoven R, Cioffi C, Ecoffet C, Gervitz L, Ionescu L, Peterson L, Fleischmann R (2016). Head-to-head comparison of certolizumab pegol versus adalimumab in rheumatoid arthritis: 2-year efficacy and safety results from the randomised EXXELERATE study. Lancet.

